# Supplementary material for: Hybrid Models and Biological Model Reduction with PyDSTool
Source: PLoS Comput Biol. 2012 Aug 9;8(8):e1002628. doi: 10.1371/journal.pcbi.1002628 (PMC3415397; doi:10.1371/journal.pcbi.1002628)
Supplement: Text S4 — Complete source code for the PyDSTool package (version 0.88.120504). Includes API documentation and help files linking to web pages. This file is identical to the current public release on Sourceforge.net. (ZIP) [file pcbi.1002628.s004.zip › PyDSTool/html/PyDSTool.Toolbox.ActivationFuncs-pysrc.html]

xml version="1.0" encoding="ascii"?


PyDSTool.Toolbox.ActivationFuncs


| Home | Trees | Indices | Help | PyDSTool |
| --- | --- | --- | --- | --- |

Package PyDSTool ::
Package Toolbox ::
Module ActivationFuncs

[hide private]

[frames] | no frames]

# Source Code for Module PyDSTool.Toolbox.ActivationFuncs

```
 1  from PyDSTool import * 
 2  from PyDSTool.Toolbox import * 
 3  from time import clock 
 4   
 5  # -------------------------------------------------------------------------- 
 6  # Helper functions 
 7  # -------------------------------------------------------------------------- 


8 -def Sigma(vo, theta, k):


9      return 1.0 / ( 1.0 + Exp( ( vo - theta ) / k ) )

10   


11 -def Sigma2(vo, a, b):


12      return 1.0 / ( 1.0 + Exp( -( a + b*vo  ) ) )

13   
14   


15 -def SigmaNeg(vo, theta, k):


16      return 1.0 / ( 1.0 + Exp( -( vo + theta ) / k ) )

17   


18 -def Beta(vo, theta, k, c):


19      return c / ( Cosh((vo-theta)/(2*k)) )

20   


21 -def SigmaV(vo, a, b, c, d, e):


22      return ((a*v0 - b)/(c*(1-Exp(-(d+e*v0)))))

23   


24 -def Gamma(vo, a, b, c):


25      return (a * Exp(-(b+c*vo)))

26
```

  


| Home | Trees | Indices | Help | PyDSTool |
| --- | --- | --- | --- | --- |

|  |  |
| --- | --- |
| Generated by Epydoc 3.0.1 on Fri May 4 15:24:18 2012 | http://epydoc.sourceforge.net |
